# Specificity of Biogenic Selenium Nanoparticles for Prostate Cancer Therapy With Reduced Risk of Toxicity: An *in vitro* and *in vivo* Study

**DOI:** 10.3389/fonc.2019.01541

**Published:** 2020-01-17

**Authors:** Praveen Sonkusre

**Affiliations:** Institute of Microbial Technology, Chandigarh, India

**Keywords:** biogenic selenium nanoparticles, necroptosis, LNCaP-FGC, tumor necrotic factor, androgen receptor, interferon regulatory factor, prostate-specific antigen, L-selenomethionine

## Abstract

Selenium deficiency is associated with many physiological disorders including the high risk of cancer. The rehabilitation of selenium with different selenium supplements, however, fails due to their low therapeutic index. Therefore, it is advantageous to have a less toxic form of selenium for supplementation with potentially high anticancer activity. Here we show *Bacillus licheniformis* derived biogenic selenium nanoparticles at a minimal concentration of 2 μg Se/ml induce necroptosis in LNCaP-FGC cells, without affecting the RBC integrity. Real-time gene expression analysis indicated the overexpression of tumor necrotic factor (*TNF*) and interferon regulatory factor (*IRF1*) and decreased expression of androgen receptor (*AR*) and prostate-specific antigen (*PSA*). Furthermore, histopathological analysis showed the subsequent oral administrations of 10 times higher concentration of these endotoxin free selenium nanoparticles in C3H/HeJ mice (50 mg Se/kg of body weight), induce significantly lower toxicity compared to the L-selenomethionine (5 mg Se/kg). Our study suggested that the biogenic SeNP could emerge as the safest form of selenium supplementation with potent anticancer activity.

## Introduction

Cancer is the leading cause of mortality in both developed and underdeveloped countries. The number of incidences is expected to rise due to the changes in the lifestyle. The major factors responsible for this are physical inactivity, obesity, changed eating habits and reproductive patterns (1). Prostate cancer is one of the common devastating diseases in males with the highest morbidity rate in the developed countries and acquires second position worldwide (1, 2).

Selenium is an essential micronutrient with well documented anticancer potential. Almost every form of selenium has an anticancer property with a diverse mechanism of action. The variation in their mechanism is largely influenced by the selenium species and the presence of associated moieties (3–7). Despite this, most of the selenium compounds have failed to come up as a chemotherapeutic agent due to the dose-related adverse toxicity. The low therapeutic index of selenium compounds also leads to selenium toxicity in selenium-deficient individuals during supplementation with a higher dose (8–10). In such cases, blood selenium levels shoot up rapidly and cause various deleterious effects. Therefore, an efficacious and less toxic form of selenium is highly desired for selenium based cancer chemotherapy and/or supplementation.

Toxicity studies on laboratory animals with different seleno-compounds suggested the lowest toxicity and improved bioavailability of chemically synthesized selenium nanoparticles (SeNPs) compared to other seleno-compounds. These particles also have similar potential of increasing the activity of selenoenzymes like glutathione peroxidases and thioredoxin reductases compared to commercially available selenium supplements like Se- methylselenocysteine and L- selenomethionine (SeMet) (6, 7, 11).

Due to the advantage of more biocompatibility and better stability of biogenic particles over chemical synthesis, we considered biogenic SeNP as the most suitable candidate for dietary selenium supplementation. The greater biocompatibility and better stability of these particles are reported to be due to the differential nanostructural arrangements of selenium atoms (12). Some biogenic SeNPs are also reported to induce cell death in various cancer cell lines including Hep-G2, MCF-7, 4T1, and HeLa (13–15). However, their effect was observed at higher doses. Also, the *in vivo* toxicity of these particles has not been explored. Therefore, we have investigated the *in vitro* anticancer activity and *in vivo* toxicity of *Bacillus licheniformis* derived SeNPs on human prostate epithelial carcinoma cells, LNCaP, and in C3H/HeJ mice model respectively.

An excellent necroptosis-inducing ability was observed by these 110 nm sized spherical SeNPs in LNCaP cells via the activation of tumor necrotic factor (*TNF*) and interferon regulatory factor (*IRF1*) at a minimum concentration of 2 μg Se/ml. Further, the treatment of SeNP reduces the expression of androgen receptor (*AR*) and prostate-specific antigen (*PSA*), the key players of the initiation and progression of prostate cancer (16, 17). Moreover, oral administration of SeNP in C3H/HeJ mice showed lower liver selenium accumulation and lower toxicity compared to the SeMet. Here we have reported that the biosynthetic SeNPs are an effective anticancer agent with lesser toxicity.

## Materials and Methods

### Synthesis of Selenium Nanoparticles

**Figure 1:**
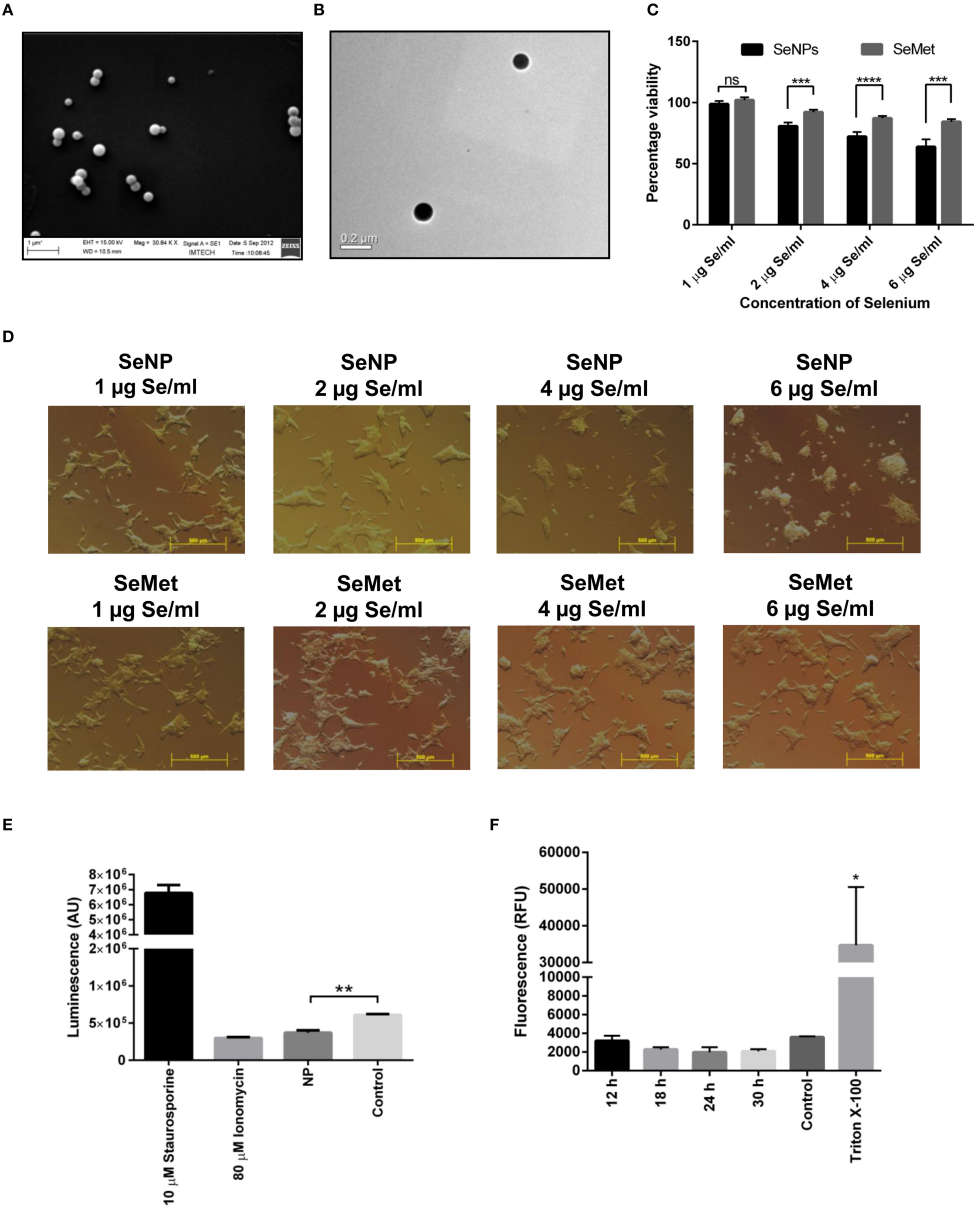
Effects on LNCaP cell viability upon treatment with different concentrations of SeNP or SeMet. **(A)** SEM and **(B)** TEM images of extracted and purified biogenic SeNPs from *Bacillus licheniformis* JS2 strain. **(C)** XTT assay showing the percentage viability of LNCaP cells after 24 h treatment with SeNPs or SeMet at a concentration of 1, 2, 4, and 6 μg Se/ml with respect to the untreated cells. A significant decrease in the cell viability of SeNP treated cells was observed compared to the SeMet treated cells with ^***^p < 0.001 and ^****^p < 0.0001 at concentrations of 2 μg Se/ml and above. The decrease in the cell viability at 2, 4, and 6 μg Se/ml SeNP was also statically significant with ^**^p-value < 0.01 compared to 1 μg Se/ml SeNP. The experiment was conducted in triplicates. **(D)** Bright field microscopy images were taken at 40 X magnification representing the morphological changes in LNCaP cells after 24 h of treatment with SeNP or SeMet at selenium concentrations of 0–6 μg/ml. **(E)** Caspase-3/7 activity was measured in LNCaP cells treated with 2 μg Se/ml SeNP by using ApoTox-Glo assay kit containing a luminogenic caspase-3/7 substrate. The measurement is based on the caspases mediated cleavage of the substrate resulting in a “glow-type” luminescent signal. The amount of luminescent signal is proportional to the caspases activity in the cells. The experiment was performed in triplicate. ^**^p < 0.01 indicates a significant difference in the caspase-3/7 activity between SeNP treated and untreated LNCaP cells. **(F)** Cells treated for 6, 12, 18, 24, and 30 h with 2 μg Se/ml SeNPs showed no release of LDH in the culture medium compared to the PBS treated cells (negative control). Cells treated with Triton-X 100 were taken as positive control. The experiment was performed in triplicate. ^*^p < 0.05 represents a significant difference between the LDH release from the positive control and SeNP treated cells.

Intracellular selenium nanoparticles were synthesized and extracted from *Bacillus licheniformis* JS2 strain under 1.8 mM sodium selenite stress as reported previously (Figures 1A,B) (18).

### Reagents

Tryptic soya broth and agar were purchased from Hi-Media Laboratories. Lysozyme, Sodium dodecyl sulfate (SDS), absolute ethanol, necrostatin-1, XTT sodium salt [2,3-bis(2-methoxy-4-nitro-5-sulfophenyl)-2H-tetrazolium-5-carboxanilide], MTT [3-(4,5-Dimethyl-2-thiazolyl)-2,5-diphenyl-2H-tetrazolium bromide], menadione, staurosporine solution, ionomycin, glutaraldehyde, paraformaldehyde, and Triton X-100 were procured from Sigma-Aldrich. Tris-buffer, 1-octanol, HCl, and chloroform, were obtained from Merck-Millipore. RPMI medium 1640, HEPES buffer, penicillin-streptomycin solution (Pen-Strep), fetal bovine serum (FBS), and TRIzol Reagent were purchased from Gibco-Invitrogen. L-selenomethionine was purchased from Calbiochem. ApoTox-Glo Triplex assay kit for measuring Caspase3/7 levels and CytoTox-ONE™ Homogeneous Membrane Integrity Assay kit for LDH release assay were purchased from Promega and the manufacturer's protocol was followed. Verso cDNA Synthesis Kit and DyNAmo ColorFlash SYBR Green qPCR kit were obtained from ThermoFisher Scientific. Limulus Amebocyte Lysate (LAL) reagent and standard endotoxin were purchased from Lonza. All plastic ware for cell culture was purchased from Nunc. Type I Millipore water was used in all the experiments.

### Quantification of Selenium

A 3:1 solution of nitric acid: perchloric acid was used for the overnight digestion of SeNPs. Digested samples were analyzed for quantification of selenium in a Shimadzu AA-6800 atomic absorption spectrophotometer (AAS). Selenium was detected at 196 nm using the selenium cathode lamp and air-acetylene oxidizing flame.

### Cell Lines and Cell Culture

A human prostate epithelial carcinoma cell line; Derived from metastatic site: left supraclavicular lymph node (LNCaP-FGC), purchased from ATCC, was gifted by Dr. G.P.S. Raghava, Indraprastha Institute of Information Technology, Delhi, India. Cells were grown in RPMI 3160 medium supplemented with HEPES buffer (10 mM), penicillin and streptomycin solution (100 units and 50 units/ml respectively), and fetal bovine serum (10%), at 37°C in a humidified incubator with 5% CO_2_.

### Cell Viability Assay

LNCaP cells were seeded in a 96-well flat bottom Nunclon Delta surface cell culture plate at a density of 5 × 10^3^ cells per well in RPMI 3160 medium supplemented with 10 mM HEPES buffer, antibiotics, and 10% FBS. After 24 h of resting period at 37°C, cells were treated with and grown in the presence of 1–6 μg Se/ml SeNP or SeMet for 24 h. Cell viability was determined under SeMet and SeNP stress using XTT solution as reported previously (18). Morphological changes in the presence of various concentration of selenium were also visualized under an Olympus IX71 bright field microscope using 40 X optical lens.

### Caspase-3/7 Activity

LNCaP cells were seeded in a white colored 96-well opaque walled Nunclon Delta surface cell culture plate at a density of 8 × 10^3^ cells per well in RPMI 3160 medium supplemented with 10 mM HEPES buffer, 10% FBS, and antibiotics. Levels of activated caspase-3/7 were detected after 12 h treatment with 2 μg Se/ml SeNP as per our previously reported protocol using ApoTox-Glo Triplex assay kit (18).

### Lactate Dehydrogenase (LDH) Release Assay

Rupture of the cell membrane and removal of intracellular components to the surrounding medium is a hallmark feature of necrosis. The release of LDH in the medium after SeNP treatment was determined using CytoTox-ONE^TM^ Homogeneous Membrane Integrity Assay Kit (Promega). LNCaP cells were seeded in a white colored 96-well opaque walled Nunclon Delta surface cell culture plate at a density of 1 × 10^3^ cells per well in RPMI 3160 medium supplemented with 10 mM HEPES buffer, 10% FBS, and antibiotics. After 24 h of resting period at 37°C in a 5% CO_2_ incubator, 2 μg Se/ml SeNPs were added to the test wells and the plate was further incubated for 12, 18, 24, or 30 h at 37°C. CytoTox-ONE^TM^ assay kit was used according to the manufacturer's protocol. The fluorescent signals corresponding to the LDH release was estimated on a BioTek Power Wave Microplate reader.

### RNA Extraction, cDNA Synthesis, and Real-Time PCR

LNCaP cells were seeded in 6-well Nunclon Delta surface cell culture plates at a density of 5 × 10^5^ cells per well in RPMI medium supplemented with 10 mM HEPES buffer, 10% FBS and antibiotics. Plates were incubated for 24 h at 37°C. SeNPs at a concentration of 2 or 4 μg Se/ml were added to the test wells and incubated further for 16 h. RNA extraction, cDNA synthesis, and Real-Time PCR were performed as per our previously reported protocol (19).

### Necroptosis Inhibition

Cells were seeded in RPMI 3160 medium at a density of 5 × 10^3^ cells per well in 96-well flat bottom Nunclon Delta surface cell culture plates. After 24 h of resting period at 37°C in a 5% CO_2_ incubator, cells were treated with DMSO or 2 μg Se/ml SeNPs or 2 μg Se/ml SeNPs along with necrostatin-1 (Nec-1) at three different concentrations viz., 20, 50, or 100 mM and cultured for another 24 h at 37°C. MTT assay for cell viability was performed as per our previously reported protocol (19).

### Endotoxin Test

Endotoxin test was performed on the *B. licheniformis* derived SeNPs with Limulus Amebocyte Lysate (LAL) reagent. Endotoxin limit of SeNPs was determined by using the formula, Endotoxin Limit (EL) in EU/ml = maximum acceptable bacterial endotoxin/maximum daily dose. Maximum Valid Dilution (MVD) was determined by using the formula, MVD = EL × Concentration of SeNPs/λ, where λ is the sensitivity of the tested Limulus Amebocyte Lysate used (in EU/ml). Different dilutions and mathematical formulae were applied according to the manufacturer's protocol to estimate the levels of endotoxin in extracted SeNPs.

### Hemolysis Assay

Human RBCs collected from the blood of healthy volunteers were washed three times with PBS (pH 7.4). Cells were resuspended, diluted and distributed equally in 8 different vials and treated with 6 different concentrations of SeNPs viz. 1, 2, 4, 6, 50, or 200 μg Se/ml. Samples were mixed briefly and kept undisturbed at 37°C. 1% Triton × 100 treated RBCs were used as positive control. Whereas, PBS treated RBCs were taken as a negative control. Samples were withdrawn from each tube after 4, 12, and 24 h of incubation and centrifuged at 1,000 × g for 10 min at RT. The supernatant was collected in a 96 well flat bottom plate and read at 414 nm on a BioTek Power Wave Microplate reader for hemoglobin release. Experiments were performed in triplicate.

### Animal Experimentation and Ethical Statement

Male C3H/HeJ mice were bred in-house at the animal house facility at Institute of Microbial Technology. Approval for the animal experiments was granted by the Institutional Animal Ethical Committee and performed according to the National Regulatory guidelines issued by the Committee for the Purpose of Control and Supervision of Experiments on Animals (registration no. 55/1999/CPCSEA), Ministry of Environment, Forest and Climate Change, Government of India.

### Median Lethal Dose (LD_50_) Estimation of SeNP and SeMet

Eight to ten weeks old male C3H/HeJ mice were used as a subject to determine the median lethal dose. Fifty mice were randomly divided into 10 groups of five mice per group. A single acute dose of SeMet or SeNP prepared in sterile distilled water was administered orally at five different concentrations as indicated in Table 1. After the treatment, mice were kept under observed for 14 days and the number of deaths was observed to calculate LD_50_ value.

**Table 1:** Concentrations of selenium, in the form of SeMet and SeNPs, used for oral dosing in C3H/HeJ mice (*n* = 5).

**SELENIUM DOSE**	
**SeMet**	**SeNPs**
5 mg Se/kg	10 mg Se/kg
7.5 mg Se/kg	20 mg Se/kg
10 mg Se/kg	50 mg Se/kg
15 mg Se/kg	70 mg Se/kg
20 mg Se/kg	110 mg Se/kg

### Short-Term Toxicity

Fifteen C3H/HeJ mice were randomly divided into three groups of five mice per group. A solution of SeMet or SeNP prepared in sterile distilled water was administered orally to the mice. The control group had received saline, the mice in one group had received SeMet at a dose of 5 mg Se/kg and mice in another group received SeNPs at a dose of 50 mg Se/kg for 10 consecutive days.

### Blood and Tissue Collection

At the end of the experiments, blood was collected from the retro-orbital plexus of each mouse into heparinized tubes. Mice were anesthetized with diethyl ether and sacrificed by cervical dislocation; kidney, liver, and spleen were collected and fixed in 10% formalin for histopathology study. Some liver sections were collected separately and digested overnight in a 3:1:: nitric acid: perchloric acid solution. The digested samples were analyzed on graphite furnace for the quantification of liver selenium using AAS.

### Biochemical Parameters

Blood was collected from retro-orbital plexus of each mouse. The levels of alanine aminotransferase (ALT), aspartate aminotransferase (AST), creatinine, and alkaline phosphatase (ALP) in blood plasma were estimated spectrophotometrically, and the levels of triglycerides (TG) were measured enzymatically at Medicos Centre- pathology laboratory, Chandigarh, using commercially available kits.

### Histopathology

To determine the selenium-induced tissue toxicity, different organs of mice (kidney, liver, and spleen) were processed for hematoxylin and eosin (H&E) staining at Medicos Centre, Chandigarh. Slides were examined under a light microscope.

### Statistical Analysis

All the experiments were performed in triplicates and represented here as mean ± SD unless otherwise mentioned. Statistical significances were examined on GraphPad prism6 software using unpaired Student's *t*-test.

## Results

### Biogenic SeNPs Decreases the Cell Viability Independent to Apoptosis and Necrosis

Initial screening for anticancer activity was performed with an XTT assay. LNCaP cells were exposed to different concentrations of SeNP or SeMet for 24 h. A significant decrease in the cell viability was observed in cells treated with SeNP at a concentration of 2 μg Se/ml and above. The viability was decreased with the increase in the concentration of SeNP and it was only 60% at a dose of 6 μg Se/ml. A significant difference between the activity of SeNP and SeMet was also observed at all the concentrations tested at and above 2 μg Se/ml (Figure 1C).

In support of the XTT results, bright field micrographs also confirmed the cellular damage upon SeNP treatment. Most of the cells were detached from the surface, shrunken and lost their typical morphology. Rest of the cells aggregated and formed big clumps on the plate surface. The morphological changes were directly proportional to the concentration of SeNPs added. Whereas, no significant change was observed in SeMet treated cells (Figure 1D).

To determine apoptosis, levels of caspase-3/7 were estimated in SeNP treated LNCaP cells using ApoTox-Glo triplex assay kit. A significant decrease in the luminescence signal was observed after 12 h of treatment with 2 μg Se/ml SeNP, suggested low caspase activity in these cells compared to the untreated cells. Results indicated that the SeNPs are inducing cytotoxicity through a caspase-independent pathway (Figure 1E).

Necrosis is always associated with the loss of membrane integrity and thus release of lactate dehydrogenase (LDH) in the surrounding medium. However, no significant increase in the LDH release was observed in LNCaP cells treated with 2 μg Se/ml SeNP for 12, 18, 24, and 30 h compared to the untreated cells (Figure 1F). Results suggest that SeNP induces cell death neither through apoptosis nor necrosis.

### SeNP Induces the Expression of Necroptosis Associated Genes in LNCaP Cells

**Figure 2:**
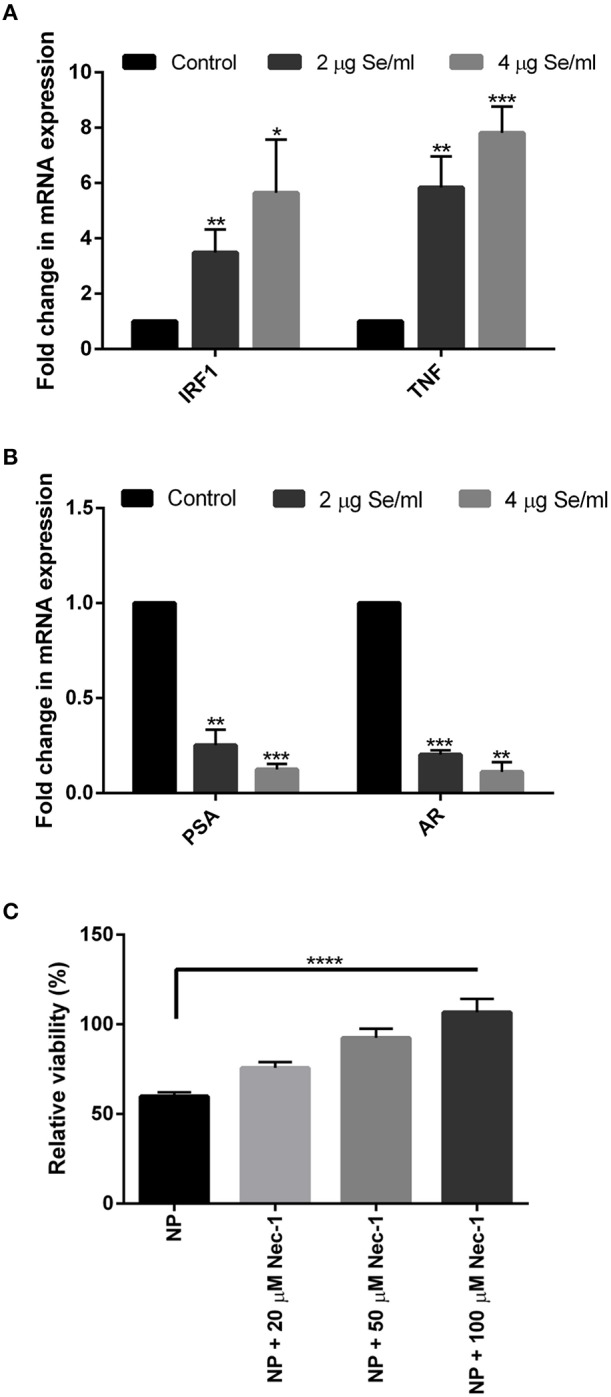
**(A,B)** Real-time gene expression profiling of SeNPs treated and untreated LNCaP cells was performed. Significant dose-dependent fold change (^*^p < 0.05, ^**^p < 0.01, and ^***^p < 0.001) was observed in the expression of *IRF1, TNF, PSA*, and *AR* gene in LNCaP cells on SeNP treatment. The experiment was conducted in triplicate. **(C)** LNCaP cells were cultured in the presence of 2 μg Se/ml SeNPs along with 20, 50, or 100 μM Necrostatin-1 for 24 h. Cell viability determined with MTT assay showed more viable cells in wells treated with SeNP along with Necrostatin-1 compared to SeNP treated cells. Cell viability was directly proportional to the concentration of Necrostatin-1 used. The results were confirmed by three repetitions of experiments and each experiment was conducted in triplicate. ^****^p < 0.0001 represents a significant difference in the viability of LNCaP cell treated with SeNP or SeNP with 100 μM Necrostatin-1.

To further validate the above results and to explore the cell death pathway, real-time mRNA expression analysis of signature genes from various cell death pathways was performed. Real-time data showed a dose-dependent overexpression of the necroptosis associated *TNF* and *IRF1* mRNA in SeNP treated cells. More than 3 folds and 5 folds increase was observed in the expression of these genes under 2 and 4 μg Se/ml SeNP treatment, respectively (Figure 2A). Contrary to this, more than 70 and 80% decrease in the expression of both *PSA* and *AR* was also observed under 2 and 4 μg Se/ml SeNP stress, respectively (Figure 2B).

An MTT assay was performed to determine the viability of SeNP treated cells cultured in the absence or presence of Necrostatin-1 (Nec-1), a potent necroptosis inhibitor. A significant increase in the relative cell viability was observed in Nec-1 supplemented wells. The viability of cells was proportional to the concentration of Nec-1 added (Figure 2C).

### SeNPs Are Free From Bacterial Endotoxins

**Figure 3:**
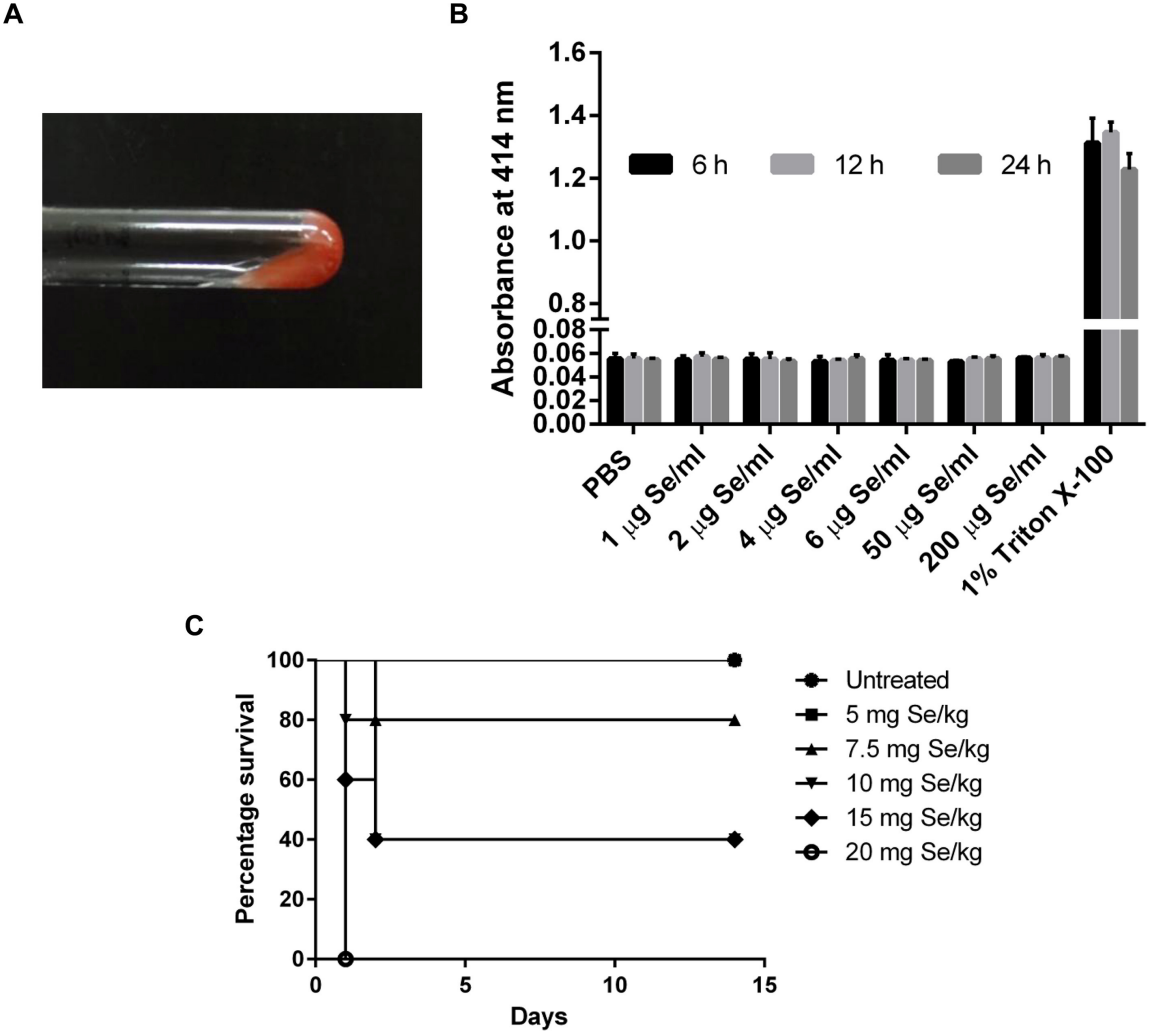
**(A)** LAL gel clot assay showing no clot formation in the tested SeNP samples. The image is representative of all the concentration tested. **(B)** Absorbance at 414 nm indicated no hemolysis of RBC upon treatment with different concentrations of SeNPs for 6, 12, and 24 h. The graph is the representation of three independent experiments. **(C)** Percentage survival of C3H/HeJ mice subjected to the oral dosing of different concentrations of L-selenomethionine.

The presence of endotoxin was tested using LAL reagent in a gel-clot assay with an endotoxin detection limit of 0.06 EU/ml where the formation of a gel clot was the indicator of the presence of endotoxin in the sample. No clot formation was observed at all the concentrations tested, suggested the absence of endotoxins in SeNP sample (Figure 3A).

### SeNPs Do Not Affect the RBC Integrity

Hemolysis assay was performed to explore the cytotoxic effect of SeNPs on human RBCs. Human erythrocytes were exposed to SeNPs at a concentration ranging from 1 to 200 μg Se/ml for different time intervals, up to 24 h. No hemoglobin release was observed in the surrounding medium suggesting the non-deleterious effect of SeNPs on RBC integrity, even at the highest dose of 200 μg Se/ml (Figure 3B).

### SeNP Induce Minor Toxicity in Mice Compared to SeMet

#### Median Lethal Dose

A comparative study was performed to estimate the LD_50_ of SeNP and SeMet in C3H/HeJ mice. No mortality was observed in mice received 5 mg Se/kg SeMet. However, all the 5 mice died within the first 24 h of dosing with 20 mg Se/kg SeMet. Interestingly, oral administration of SeNPs did not show mortality at any of the concentration tested. The data of acute toxicity in the form of a number of deaths per day and percentage mortality are shown in Tables 2, 3 respectively. From the observations, the LD_50_ value for selenium in the form of SeMet was calculated as 11.826 mg Se/kg body weight in C3H/HeJ mice. Whereas, the LD_50_ value of SeNPs cannot be determined. The survival curve of SeMet treated mice is shown in Figure 3C.

**Table 2:** Mortality observed in C3H/HeJ mice after a single acute dose of L-selenomethionine (SeMet) or SeNP.

**Days**	**Number of deaths at various doses of SeMet**				
	**5 mg Se/kg**	**7.5 mg Se/kg**	**10 mg Se/kg**	**15 mg Se/kg**	**20 mg Se/kg**
1	0	0	1	2	5
2	0	1	2	1	0
3	0	0	0	0	0
4	0	0	0	0	0
5	0	0	0	0	0
6	0	0	0	0	0
7	0	0	0	0	0
8	0	0	0	0	0
9	0	0	0	0	0
10	0	0	0	0	0
11	0	0	0	0	0
12	0	0	0	0	0
13	0	0	0	0	0
14	0	0	0	0	0
**Days**	**Number of deaths at various doses of SeNPs**				
	**10 mg Se/kg**	**20 mg Se/kg**	**50 mg Se/kg**	**70 mg Se/kg**	**110 mg Se/kg**
1	0	0	0	0	0
2	0	0	0	0	0
3	0	0	0	0	0
4	0	0	0	0	0
5	0	0	0	0	0
6	0	0	0	0	0
7	0	0	0	0	0
8	0	0	0	0	0
9	0	0	0	0	0
10	0	0	0	0	0
11	0	0	0	0	0
12	0	0	0	0	0
13	0	0	0	0	0
14	0	0	0	0	0

*The lethal effect was observed only in the initial days of the SeMet treatment and no death was observed after day 2. Again, no mortality was observed in the group of SeNP treated mice*.

**Table 3:** Percentage mortality in different groups of mice subjected to different doses of selenium.

**SeMet**		**SeNPs**	
**Selenium dose**	**Mortality (%)**	**Selenium dose**	**Mortality (%)**
5 mg Se/kg	0	10 mg Se/kg	0
7.5 mg Se/kg	20	20 mg Se/kg	0
10 mg Se/kg	60	50 mg Se/kg	0
15 mg Se/kg	60	70 mg Se/kg	0
20 mg Se/kg	100	110 mg Se/kg	0

#### Short-Term Toxicity and Blood Parameter Analysis

**Figure 4:**
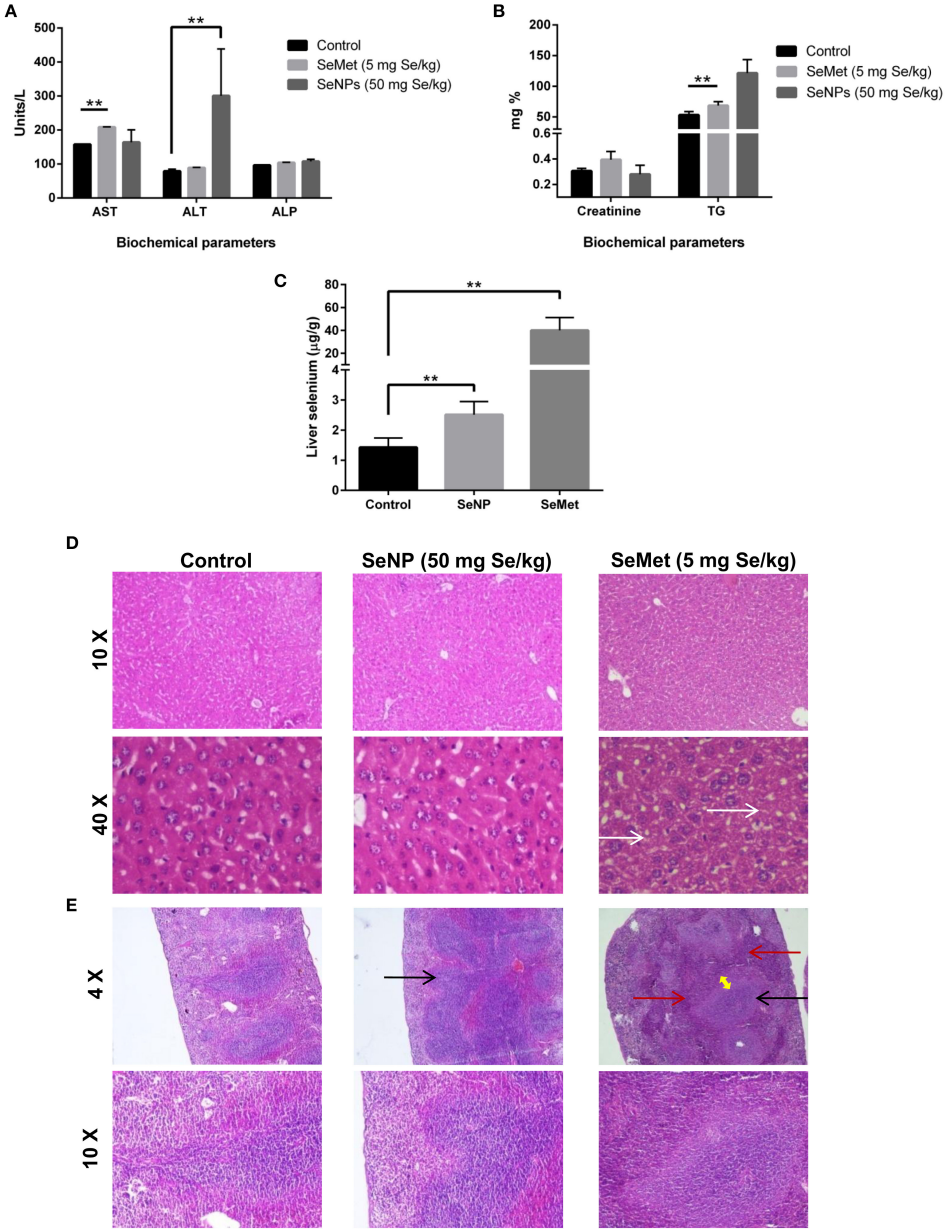
**(A,B)** Liver and renal function tests were performed to determine the selenium-induced toxicity in C3He/J mice. Levels of AST, ALT, Creatinine, ALP, and TG were estimated in the blood plasma of SeMet or SeNP treated mice with a selenium concentration of 5 and 50 mg/kg of body weight respectively. A significant increase (^**^p > 0.01) was observed in the levels of AST and TG in SeMet treated mice compared to the control, whereas, increase in the levels of ALT was observed upon SeNP treatment. Statistical significance was determined using Two-way ANOVA. **(C)** Accumulation of selenium in the liver was estimated using AAS in a graphite furnace mode. A significant increase in the liver selenium was observed in both the SeNP and SeMet treated mice compared to the control. However, the concentration was more than 25 times in the case of SeMet compared to the 2 times increase in case of SeNP with respect to the control. **(D)** H&E stained liver sections of control, SeNP, and SeMet treated mice. Specimens visualized at 10X and 40X showing a fatty change (steatosis) in scattered liver cells of SeMet treated mice (indicated by white arrow), whereas, no injury or morphological alteration was observed in the tissue specimen of SeNP treated mice compared to the control. **(E)** H&E stained spleen sections of control, SeNP, and SeMet treated mice. A specimen of SeNP treated mice visualized at 4X and 10X showing non-follicular reactive spleen with enlargement of the lymphoid tissue (indicated by black arrow). Whereas, reactive lymphoid follicular hyperplasia (black arrow) and the infiltration of lymphocytes to the red pulp area (red arrow) with the development of a marginal zone (yellow arrow) was observed in spleen section of mice treated with SeMet. The experiment was conducted in triplicate.

The levels of aspartate aminotransferase (AST), alanine aminotransferase (ALT), creatinine, alkaline phosphatase (ALP), and triglycerides (TG) were estimated in the blood plasma of mice treated with placebo, SeMet or SeNPs for 10 consecutive days. Results showed significant toxicity in both the cases compared to the placebo. SeMet treatment showed a significant increase in the circulating levels of AST and TG. Nevertheless, SeNP treatment showed an increase in the levels of ALT and TG. However, the increase in TG was not statistically significant (Figures 4A,B). AAS analysis showed more than 25 times accumulation of selenium in the liver sections of SeMet treated mice. Conversely, only twice the concentration of selenium was present in SeNP treated group compared to control (Figure 4C).

#### Histopathology

H&E stained tissue sections showed non-follicular reactive spleen with the enlargement of lymphoid tissue in SeNP treated mice. In contrast, reactive lymphoid follicular hyperplasia with the development of a marginal zone was observed in the spleen section of SeMet treated mice. In addition, microvesicular fatty change (steatosis) was also observed only in the liver tissue of SeMet treated mice (Figures 4D,E). No change was observed in the kidney tissue architecture in both the cases, compared to the control (data not shown).

## Discussion

At higher doses, selenium is reported as an excellent anticancer agent, specifically for prostate, lung, breast, and colon cancers. However, due to the low therapeutic index, a marginal increase in the blood selenium level causes severe toxicity (3, 20). Therefore, it is essential to maintain an adequate amount of selenium in the body to treat cancer without inducing toxicity. Alternatively, it will be beneficial to utilize a less toxic form of selenium to fulfill the requirement. Reports on chemically synthesized SeNP suggested lower toxicity and equivalent anticancer activity compared to inorganic and organic selenium compounds (3, 11, 21, 22). Similarly, biogenic SeNPs synthesized by *Acinetobacter, Streptomyces*, and *Halococcus* also showed anticancer activity, however, at higher doses (13–15). Earlier in our study, we have shown that a concentration of 2 μg Se/ml of our sterically stabilized biogenic SeNPs is very effective in inducing *TNF*/*IRF1* mediated necroptosis in PC-3 cells without compromising the viability of human peripheral blood mononuclear cell (hPBMC) (18, 19).

In this part of the study, the effect of biogenic SeNPs on human prostate epithelial carcinoma cells, LNCaP was investigated. The result showed a significant decrease in the viability of LNCaP cells at the same minimum concentration of 2 μg Se/ml. We also observed a significant decrease in the activity of caspase 3/7, the key players of the apoptotic pathway, and no LDH release from the cells, the signature feature of necrosis. To dig into more detail, real-time gene expression analysis of characteristic genes of different cell death pathways showed over-expression of the only necroptosis associated *TNF* and *IRF1* genes and down-regulation of *AR* and *PSA* in these cells. The over-expression of *TNF* and *IRF1* was found to be similar to our earlier observation in PC-3 cells (19). Further, the inhibition of SeNP induced cell death under the presence of necrostatin-1, a potent necroptosis inhibitor, confirmed the cell death pathway is necroptosis.

*AR* is usually expressed in most of the prostate cancer cells and plays differential roles in cell growth, proliferation and development of both androgen dependent and androgen independent cancer (23, 24). Whereas, *PSA* codes for a serine protease downstream to the *AR*. Targeting AR is reported to induce apoptosis, autophagic cell death and programmed necrosis (25, 26) and it is currently used as a constructive approach to inhibit prostate cancer cell proliferation. Different attempts have been made to target *AR* expression and signaling for the treatment of prostate cancer. For example, inhibition of AR protein expression using antisense oligodeoxynucleotides which bind to its complementary target mRNA and suppress its protein synthesis (27). In another approach, the activity of androgen receptor was disrupted using Anti-AR antibody and an *AR* mRNA hammerhead ribozyme (28). Antiandrogens, androgen biosynthesis inhibitors, and siRNA were also used to target AR (29, 30). Similar to the current approach of inhibiting prostate cancer by targeting AR we have also observed the SeNP induced down-regulation of *AR* and the induction of cell death. Collectively, our results showed that these biogenic SeNPs has the ability to inhibit the growth of prostate cancer cell through the activation of *TNF* and *IRF1* and the suppression of *AR*. Here, we hypothesize that probably these two events are either interconnected (31, 32) with an unexplored pathway or they are acting differently to induce necroptosis.

Prior to animal experiments, the presence of NP associated bacterial endotoxins was determined. Endotoxin is invariably associated with lipopolysaccharide fraction of Gram-negative bacteria. Lipid A component of the outer membrane is responsible for toxicity, whereas, polysaccharide part is responsible for immunogenicity. The peptidoglycan of some Gram-positive bacteria is also reported to show LPS like action during infection. However, peptidoglycans are less potent than LPS. Results of LAL reagent assay showed the absence of bacterial endotoxins in these *Bacillus licheniformis* derived SeNPs suggesting the NPs are free from endotoxins. The particles were also found to be non-toxic to RBCs even at the highest tested concentration of 200 μg Se/ml.

We tried to estimate and compare the LD_50_ of SeNPs with SeMet in C3H/HeJ mice. The LD_50_ value of selenium in the form of SeMet was calculated as 11.826 mg Se/kg body weight. However, we could not identify the LD_50_ for biogenic SeNPs. We observed no mortality even at the highest tested SeNP dose of 110 mg Se/kg body weight. Due to the experimental limitations, it was not possible to administer a SeNP dose over 110 mg Se/kg orally to the mice at a time (Table 1). The possible reason behind the survival of all the mice in this group could be (i) either the SeNPs are non-toxic to the normal cells, as we have reported earlier in case of peripheral blood mononuclear cells (18) and observed in RBCs or, (ii) in the biological system these NPs lacks absorption from the alimentary canal. According to literature selenium is reported to localize mainly in the liver where it contributes approximately 30% of the total tissue selenium (33). So, to rule out the second possibility short-term toxicity testing was performed. The increase in the total liver selenium and the plasma levels of liver enzymes upon SeNP treatment indicated the absorption of particles and minor liver toxicity. However, the absorption was significantly lower compared to SeMet. H&E stained sections of liver, kidney, and spleen of mice challenged with 50 mg Se/kg SeNP for 10 days showed no toxicity or morphological changes in liver and kidney tissue. Although, the enlargement of lymphoid tissue was observed in the spleen section compared to the control. In contrast, mice treated with SeMet at a concentration of 5 mg Se/kg were most affected. Reactive lymphoid follicular hyperplasia with the development of marginal zone and the infiltration of lymphocytes to the red pulp area was observed in the spleen tissue. Enlargement of spleen lymphoid tissue is an indicator of the hyper-reactive spleen that could be due to the immunogenic response against toxins, bacterial/viral infection or autoimmune disease. In some patients, the etiology of reactive lymphoid follicular hyperplasia cannot be ascertained. We also observed microvesicular fatty change also called fatty liver or steatosis in the hepatocytes of these mice, suggesting the accumulation of lipids in the cells. In support of this, we have also observed significantly elevated levels of total triglycerides in the blood of the same group of mice. This could be due to excess intake of lipids and/or impaired lipid metabolism. Literature suggests that selenium toxicity can inhibit thiol-containing enzymes by the incorporation of Se in place of sulfur (34). Considering this fact we proposed that steatosis could be the result of inhibition of lipid metabolizing enzymes. However, no such alterations were observed in mice treated with the same concentration of SeNP (data not shown). Results indicate that oral administration of 5 mg Se/kg SeMet is more toxic compared to the 50 mg Se/kg of biogenic SeNPs. We proposed that *Bacillus licheniformis* derived sterically stabilized SeNPs could be a potential candidate for cancer chemoprevention and therapy. Though, pharmacokinetics and immunotoxicity at therapeutic dose should be estimated.

## Conclusions

*Bacillus licheniformis* derived biogenic SeNPs are very effective in inducing prostate cancer cell death at a minimum concentration of 2 μg Se/ml through a *TNF/IRF1* mediated necroptosis pathway and by *AR* down-regulation. The two distinct pathways are either acting differently or interconnected by unexplored signaling. Additionally, we have observed the lowest SeNP induced toxicity in mice model compared to the commercially available selenium supplement and other reported bioactive selenium compounds. The study concludes that the use of *Bacillus licheniformis* derived biogenic SeNPs for cancer chemoprevention is a more successful and safe methodology compared to the earlier reports. However, *in vivo* anticancer activity, pharmacokinetics and detailed toxicity studies including cytokine profiling are required to claim this potential.

## Data Availability Statement

All datasets generated for this study are included in the article/supplementary material.

## Ethics Statement

The animal study was reviewed and approved by Institutional Animal Ethical Committee (registration no. 55/1999/CPCSEA), Ministry of Environment, Forest and Climate Change, Government of India.

## Author Contributions

PS designed and performed the experiments and prepared manuscript.

### Conflict of Interest

The author declares that the research was conducted in the absence of any commercial or financial relationships that could be construed as a potential conflict of interest.
